# Detection Rate and Clinical Impact of PET/CT with ^18^F-FACBC in Patients with Biochemical Recurrence of Prostate Cancer: A Retrospective Bicentric Study

**DOI:** 10.3390/biomedicines10010177

**Published:** 2022-01-15

**Authors:** Luca Filippi, Oreste Bagni, Carmelo Crisafulli, Ivan Cerio, Gabriele Brunotti, Agostino Chiaravalloti, Orazio Schillaci, Franca Dore

**Affiliations:** 1 Nuclear Medicine Unit, “Santa Maria Goretti” Hospital, Via Antonio Canova, 04100 Latina, Italy; o.bagni@ausl.latina.it; 2 Nuclear Medicine Unit, Azienda Sanitaria Universitaria Giuliano Isontina (ASUGI), Cattinara Hospital, Strada di Fiume 447, 34129 Trieste, Italy; carmelo.crisafulli@asugi.sanita.fvg.it (C.C.); ivan.cerio@asugi.sanita.fvg.it (I.C.); franca.dore@asugi.sanita.fvg.it (F.D.); 3 Department of Radiological Sciences, Oncology and Anatomical Pathology, Sapienza University of Rome, Viale Regina Elena 324, 00100 Rome, Italy; gabriele.brunotti@uniroma1.it; 4 Department of Biomedicine and Prevention, University Tor Vergata, Viale Oxford 81, 00133 Rome, Italy; agostino.chiaravalloti@uniroma2.it (A.C.); orazio.schillaci@uniroma2.it (O.S.); 5 IRCCS Neuromed, 86077 Pozzilli, Italy

**Keywords:** prostate cancer, PET/CT, ^18^F-FACBC, ^18^F-fluciclovine, biochemical recurrence, precision medicine, oncology

## Abstract

Our aim was to assess the detection rate (DR) of positron emission computed tomography (PET/CT) with anti-1-amino-3-[^18^F]-flurocyclobutane-1-carboxylic acid (^18^F-FACBC) in patients with biochemical recurrence (BCR) from prostate cancer (PC). As a secondary endpoint, we evaluated ^18^F-FACBC PET/CT’s impact on patients management. Clinical records of 81 patients submitted to ^18^F-FACBC PET/CT due to PC BCR in two Italian Nuclear Medicine Units were retrospectively assessed. DR was gauged in the whole cohort and stratifying patients by discrete intervals of PSA levels. PET/CT’s impact on clinical management was scored as (1) major if it entailed an intermodality change (e.g., from systemic to loco-regional therapy); (2) minor if it led to an intramodality change (e.g., modified radiotherapy field). PET/CT’s DR resulted in 76.9% in the whole cohort, with a positive predictive value of 96.7%. Stratified by PSA quartile intervals, PET/CT’s DR was 66.7%, 71.4%, 78.9% and 90% for PSA 0.2–0.57 ng/mL, 0.58–0.99 ng/mL, 1–1.5 ng/mL and >1.5 ng/mL without significant difference among groups (*p* = 0.81). The most common sites of relapse were prostate bed and pelvic lymph nodes (59.3%). PET/CT impacted on clinical management in 33/81 cases (40.7%), leading to a major change in 30 subjects (90.9%). ^18^F-FACBC PET/CT localized recurrence in patients with BCR, with meaningful DR also at low PSA levels and significantly impacted on clinical management.

## 1. Introduction

Prostate cancer (PC) is a leading cause of cancer-related death worldwide [1]. In patients with localized disease, radical treatment can be performed through prostatectomy or radiation therapy (RT). However, after a variable period of time, about 30–40% of patients present biochemical recurrence (BCR), defined, according to several parameters, as an increasing level of prostate specific antigen (PSA) following radical treatment [2,3].

Imaging plays a crucial role in BCR management, since determining whether it reflects local or distant recurrence is of utmost importance and has relevant clinical implications. In this scenario, positron emission computed tomography (PET/CT) has been successfully applied for the detection of metabolically active tumor tissue through the radiopharmaceutical choline, labeled with 18-fluorine (^18^F) or 11-carbon (^11^C) radionuclide. Radiolabeled choline represents a surrogate biomarker of phospholipids’ biosynthesis in tumor cells and has been used for the imaging of several malignancies other than PC such as multiple myeloma and hepatocellular carcinoma [4,5]. Nevertheless, specifically concerning PC, a low sensitivity of PET/CT with choline in case of low PSA levels has been reported [6]. Furthermore, the physiological retention of tracer within the bladder may hamper the detection of pelvic recurrence.

To overcome these drawbacks, several efforts have been made to develop novel tracers, characterized by higher sensitivity for the detection of relapse in patients with BCR following radical treatments.

The synthetic amino acid anti-1-amino-3-[^18^F]-flurocyclobutane-1-carboxylic acid (namely, ^18^F-FACBC, ^18^F-fluciclovine, Axumin^®^) is an L-leucine analogue showing high tumor accumulation by addressing amino acid transports, which are up-regulated in PC [7]. On the other hand, PET/CT with ligands binding to Prostate Specific Membrane Antigen (PSMA), overexpressed by PC and only minimally detectable in normal prostate tissue, has recently been introduced with overwhelming results in clinical practice, and two PSMA-targeted radiopharmaceuticals have recently been FDA approved [8,9]. However, some PSMA ligands (e.g., ^68^Ga-PSMA-11) show a meaningful accumulation in bladder; thus, the identification of loco-regional relapse might deserve particular attention and require dedicated protocols [10].

In a recently published meta-analysis encompassing 283 studies and 697 patients, ^18^F-FACBC PET/CT presented pooled sensitivity for the overall detection of recurrence of 0.68 (95% CI: 0.63–0.73), and specificity of 0.68 (95% CI: 0.60–0.75) [11]. However, the scientific data concerning the diagnostic performance of ^18^F-FACBC PET/CT in PC patients with BCR and low PSA levels are still limited. In a cohort of 126 subjects, Wang et al. reported a positivity rate of 33% at low (<1 ng/mL) and of 0% at very low (<0.3 ng/mL) PSA values [12]. More recently, Marcus et al. obtained an overall detection rate of 57.8% in patients having median serum PSA of 0.17 ng/mL, with prostate bed and pelvic lymph nodes being the most common sites of relapse [13].

Furthermore, ^18^F-FACBC PET/CT has been reported to have a relevant impact on the clinical management of patients with PC BCR, both for the choice of the most appropriate therapeutic approach (e.g., Stereotactic Body Radiation Therapy/SBRT versus systemic therapy) and the delineation of RT fields [14].

The aims of this retrospective study were: (1) to analyze the detection rate of ^18^F-FACBC PET/CT in PC patients showing BCR after surgery or RT, stratified by different PSA levels; (2) to determine PET/CT’s clinical impact on the therapeutic management of PC patients included in the analysis.

## 2. Materials and Methods

### 2.1. Study Design

We retrospectively reviewed clinical data of patients affected by PC BCR who were submitted to ^18^F-FACBC (Axumin^®^, Advanced Accelerator Applications, Isernia, Italy) PET/CT between December 2019 and July 2021 at the following Italian Nuclear Medicine Units: S. Maria Goretti Hospital, Latina, and Cattinara University Hospital ASUGI, Trieste, as a part of their diagnostic work-up.

Inclusion criteria were: (1) adult male above 18 years; (2) histologically proven PC; (3) previous prostatectomy (associated or not with lymphadenectomy and/or salvage RT) or RT/brachytherapy as a radical therapy; (4) ^18^F-FACBC PET/CT performed due to evidence of BCR, defined as a PSA of 0.2 ng/mL or higher measured more than 6 weeks after prostatectomy or a PSA rise of 2 ng/mL or higher above nadir after RT [15]. Exclusion criteria were: (1) incomplete medical history or follow-up; (2) ^18^F-FABC PET/CT performed in a clinical setting different from BCR; (3) ongoing androgen deprivation therapy (ADT).

Primary endpoint of study was to define the diagnostic performance (i.e., detection rate/DR) of ^18^F-FACBC PET/CT in all the enrolled patients. Furthermore, PET/CT’s DR for PC recurrence was assessed stratifying patients by different intervals of PSA. As a secondary endpoint, PET/CT’s impact on clinical management was assessed.

For the primary endpoint, standard of reference to finally categorize PET/CT’s results (true-positive, true-negative, false-positive, false-negative) was clinical and imaging follow-up data.

For the secondary endpoint, in each participating clinical center patients’ follow-up was analyzed together with the referring physicians during the multidisciplinary disease management team (DMT), who was asked to provide information on how clinical management was influenced by PET/CT’s results (i.e., intended therapeutic approach versus implemented therapeutic approach). In particular, PET/CT’s impact was scored as (1) major if it entailed a change in the treatment modality (intermodality change) with respect to the intended approach (e.g., from an intended ADT to an implemented SBRT), (2) minor if it led to an intramodality change (e.g., change in RT field); (3) not significant if the intended therapeutic approach was not changed.

The change management was considered to be adequate if the PSA serum level declined by more than 50% (compared to the baseline value) following treatment modification.

This was a retrospective study on data available for clinical practice in which clinical records of all patients in follow-up for PC were reviewed. Data were anonymously collected in each center and were cumulatively gathered in an electronic database for analysis. Patients were not required to give informed consent to the study because the analysis used anonymous data that were obtained after each patient agreed to being followed up and to collect clinical records by institutions. No experimental procedures, novel devices, or experimental drugs were used, and no findings were received. The study protocol conformed to the ethical guidelines of the 1975 Declaration of Helsinki.

### 2.2. ^18^F-FACBC PET/CT

All the enrolled patients underwent PET/CT with ^18^F-FACBC according to present imaging guidelines [16]. Each of the 2 centers involved in the study applied its own procedures and technology for PET/CT scan acquisition and images reconstruction, as detailed in Supplemental data 1. In brief, all patients fasted for at least 4 h before PET/CT scan and were asked to avoid any significant physical exercise 24 h prior to the scan. Whole-body PET/CT scan was performed, from the skull base to the proximal thigh, starting at 3–5 min after the injection of 370 MBq of ^18^F-FACBC.

Images were interpreted by well-trained local readers: any area with an uptake intensity greater than background uptake that could not be identified as physiological activity was considered to be potentially pathological, and its grade of uptake (maximum standardized uptake value/SUV_max_) was registered and compared with the reference value (mean standardized uptake value/SUV_mean_) measured on blood pool or bone marrow [16]. For each patient, the sites and the number of sites with pathological uptake were annotated. Indeterminate reports, unable to clearly identify the sites of recurrences, were considered to be negative for statistical analysis. To better replicate clinical practice, neither image reinterpretation nor image reading quality control between the two participating institutions was performed.

Images were then classified according to an image-based TNM staging system (PROstate cancer Molecular Imaging Standardized Evaluation/ PROMISE) originally developed for the reporting of PSMA-ligand PET/CT’s results and adapted by the authors for the reading of ^18^F-FACBC images, entailing the following regions for recurrence: prostate, prostate bed, and seminal vesicle remnants (Tr), pelvic lymph nodes (N1) (internal iliac, obturator, external iliac, perirectal, presacral, common iliac, and other), extrapelvic lymph nodes (M1a) (retroperitoneal, inguinal, chest, and other), bone (M1b), and visceral organs (M1c) [17].

### 2.3. Statistical Analysis

Data are presented as mean ± standard deviation, median, or number (percentage). Statistical analysis was performed using dedicated software (MedCalc 11.3.8.0; MedCalc Software, Mariakerke, Belgium). DR values were calculated for ^18^F-FACBC PET/CT in all patients. Fisher’s exact test was applied to examine the differences in PET/CT’s DR (primary endpoint) among different groups stratified by PSA values, significance was established at 2-tailed *p* < 0.05. Receiver Operating Characteristic (ROC) analysis was used to identify the optimal cut-off PSA for predicting a ^18^F-FACBC PET/CT positive result. Multivariate logistic regression analysis was used to estimate the association of International Society of Urological Pathology (ISUP) Grade Group, PSA and PSA doubling time (PSA DT) with positive ^18^F-FACBC PET/CT.

## 3. Results

**Figure 1:**
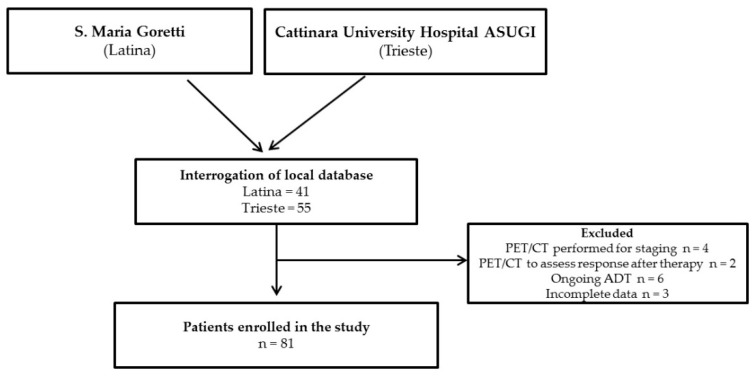
Diagnostic flow-chart for patient selection.

After the interrogation of local databases, an overall number of 96 potentially eligible patients were identified, among whom six were excluded due to ^18^F-FACBC PET/CT performed in a clinical setting different from BCR and nine due to ongoing ADT or incomplete medical history (Figure 1). An overall number of 81 patients were finally included in the study, 45 (55.5%) of whom presented BCR after prostatectomy, 20 after prostatectomy and salvage RT, 14 (18.5%) after RT and 2 (2.4%) after brachytherapy. Clinical and demographic data, encompassing age, tumor grade at diagnosis (ISUP Grade Group), initial management of prostate cancer, time from diagnosis to BCR, serum PSA prior to PET/CT, PSA DT calculated through the three most recent PSA values prior to ^18^F-FACBC PET/CT and dichotomized as < or >6 months, were recorded for all the enrolled subjects and are summarized in Table 1.

Patients’ mean age was 73.2 ± 6.9 years. The median PSA prior to PET/CT of the whole cohort resulted in 0.99 ng/mL (mean 1.27 ± 1.1 ng/mL).

Concerning ISUP grading, thirty-three patients were classified as ISUP4, twenty-seven as ISUP3, sixteen patients were ISUP1-2 and five were ISUP5.

### 3.1. Diagnostic Performance of ^18^F-FACBC PET/CT in Enrolled Patients

Among the enrolled patients, 62 (76.5%) presented ^18^F-FACBC PET/CT-positive results, while 19 (23.4%) had negative PET/CT scans. Final diagnosis was established by follow-up (n = 71, i.e., 87.7%) or follow-up and confirmatory imaging (n = 10, 12.3%). Of the 81 examined PET/CT scans, 60 were found to be true-positive, 1 true-negative, 2 false-positive, and 18 false-negative. PET/CT showed good accuracy (75.1%) for the detection of PC BCR relapse, with an overall DR of 76.9% and a positive predictive value (PPV) of 96.7%.

**Figure 2:**
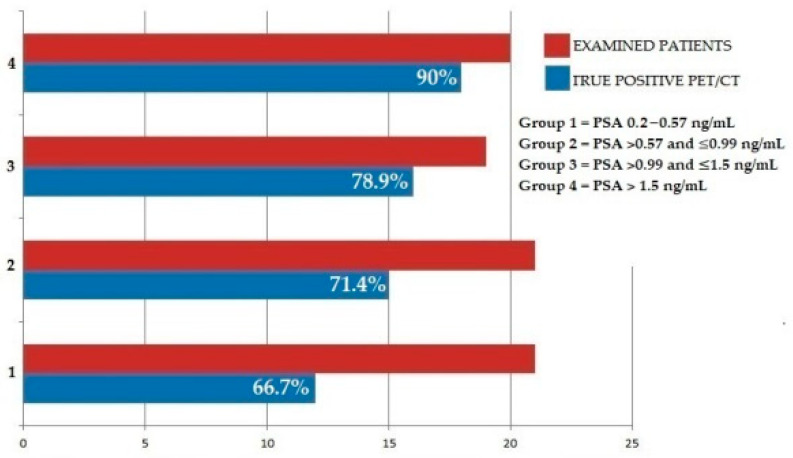
Graphic illustration of PET/CT detection rate in enrolled patients, stratified into 4 groups according to discrete PSA intervals.

PET/CT’s DR was further evaluated by stratifying patients into four different groups, according to the PSA value measured prior to ^18^F-FACBC PET/CT execution, by using discrete intervals as follows: group 1 (n = 21, PSA value between the lowest value and ≤ to the 25th percentile, i.e., 0.2 and 0.57 ng/mL), group 2 (n = 21, PSA value between the 25th and ≤ to the 50th percentile, i.e., >0.57 and ≤0.99 ng/mL), group 3 (n = 19, PSA value between the 50th percentile and ≤ to the 75th percentile, i.e., >0.99 and ≤1.5 ng/mL) and group 4 (n = 20, PSA value > to the 75th percentile, i.e., >1.5 ng). PET/CT DR was 66.7%, 71.4%, 78.9% and 90% for groups 1, 2, 3 and 4, respectively. No significant differences were found among groups (*p* = 0.81). Figure 2 summarizes PET/CT’s diagnostic performance in patients stratified by PSA intervals.

**Figure 3:**
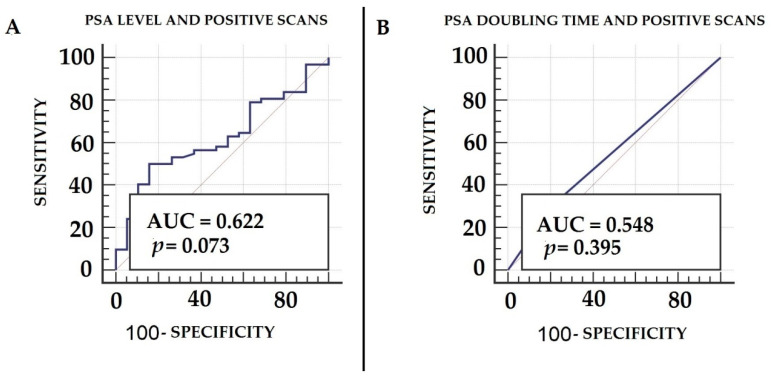
ROC curve analysis shows the accuracy of the PSA level (**A**) and PSA doubling time (**B**), measured prior to ^18^F-FACBC PET/CT scan, for predicting positive results.

**Figure 4:**
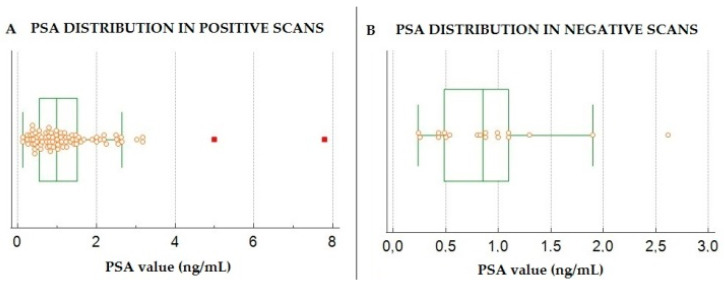
Box plot graph showing the PSA level distribution in patients with positive (**A**) and negative (**B**) ^18^F-FABC PET/CT scans, 2-tailed *t*-test results were significant at *p* = 0.005. The 2 hot dots in panel A represent 2 subjects with PSA value strongly out of range (i.e., 5 and 7.8 ng/mL, respectively).

ROC curves, generated both for PSA and PSA DT, showed the highest AUC for PSA (AUC = 0.622, 95% CI 0.5–0.72) with respect to PSA DT (AUC = 0.548, 95% CI 0.43–0.65) for predicting a PET positive scan, although neither of these parameters reached the threshold of statistical significance, with *p* = 0.07 and 0.39, respectively. At ROC analysis, the optimal cut-off value for PSA at the time of PET/CT for the prediction of a positive scan was shown to be 1.1 ng/mL (Figure 3). PSA values in patients with positive scans (mean 1.6 ± 0.8 ng/mL) were significantly higher than those measured in subjects with negative scans (mean 0.9 ± 0.59 ng/mL) at *p* = 0.005, as shown in Figure 4. Upon multivariate logistic regression analysis, including ISUP, PSA and PSA DT, no variable was significantly associated with a positive PET/CT scan (*p* = 0.19).

### 3.2. Classification of ^18^F-FACBC PET/CT’s Results According to PROMISE

Among the enrolled patients, final staging classified according to PROMISE showed positive results for local relapse (Tr/N1) in 48 patients (59.3%), detected extrapelvic lymph nodes (M1a) in eight patients (9.9%) and distant metastases to bone or visceral organs (i.e., M1b/c) in six cases (7.4%). PET/CT was negative (T0N0M0) in the remaining 19 subjects (23.4%), as shown in Table 2.

### 3.3. Impact of ^18^F-FACBC PET/CT on Clinical Management

Among the enrolled patients, change in therapeutic management occurred in 33/81 patients (40.7%). In 30 cases (90.9%), PET/CT’s clinical impact was classified as major, since it entailed an intermodality change, leading from an intended systemic therapy (androgen deprivation therapy or 2nd-generation anti-androgens) to a loco-regional PET-guided treatment consisting of SBRT on prostate bed/pelvic lymph node in 23 cases (76.6%) or on abdominal nodes in four patients (13.3%), while in three subjects (10%) the intended management plan (ADT) was revised to RT on bone lesions (M1b, n = 2) or SBRT on a lung pulmonary nodule (M1c, n = 1).

**Figure 5:**
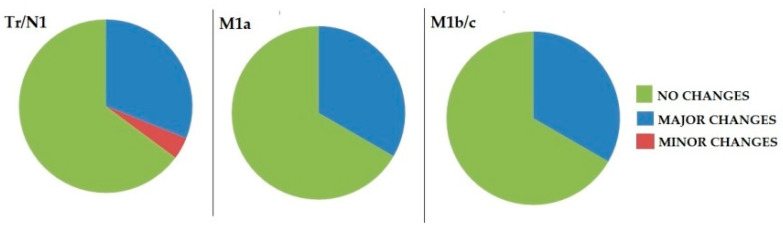
Graph illustration of the therapeutic changes determined by ^18^F-FACBC PET/CT in patients classified according to modified PROMISE reading approach.

In three patients, PET/CT presented a minor impact, since it led to the inclusion of missing ^18^F-FACBC-avid pelvic lymph nodes in the RT field. Figure 5 shows the distribution of therapeutic changes in patients classified according to PROMISE; no significant differences was found in PET/CT’s impact among groups at *p* = 0.9.

**Figure 6:**
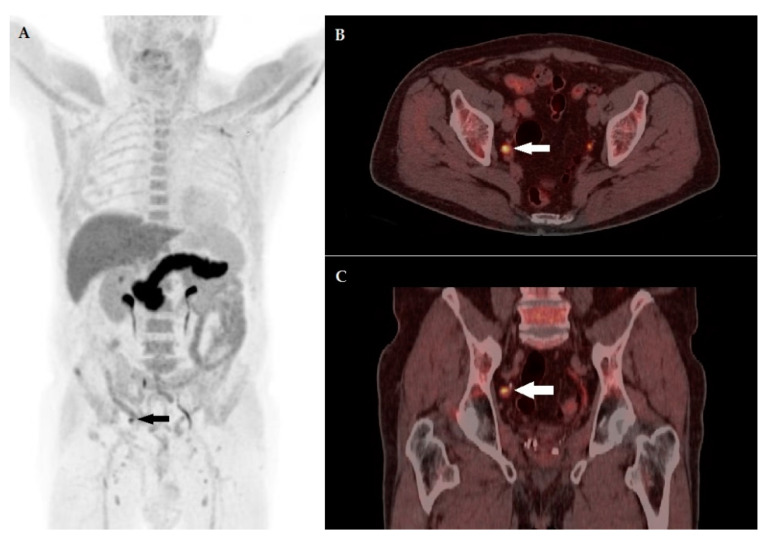
A 72-year-old patient 6 years post prostatectomy for pT3b pN0 ISUP Grade Group 4 prostate adenocarcinoma, presenting biochemical recurrence with PSA level prior to PET/CT of 0.38 ng/mL. (**A**) MIP image showing an area of increased tracer incorporation in the right pelvis (arrow). Fused corresponding PET/CT axial (**B**, arrow) and coronal (**C**, arrow) slices depicting a round-shaped right obturator lymph node, characterized by ^18^F-FACBC pathological uptake, with a maximum diameter of 12 mm and a maximum standardized uptake value (SUVmax) of 7.7, superior to bone marrow SUVmean (3.1). Final diagnosis was T0N1M0 according to PROMISE. Intended therapy prior to PET/CT was androgen deprivation therapy, after the collegial discussion of ^18^F-FACBC PET/CT’s results, the patient was submitted to stereotactic radiotherapy with PSA response.

**Figure 7:**
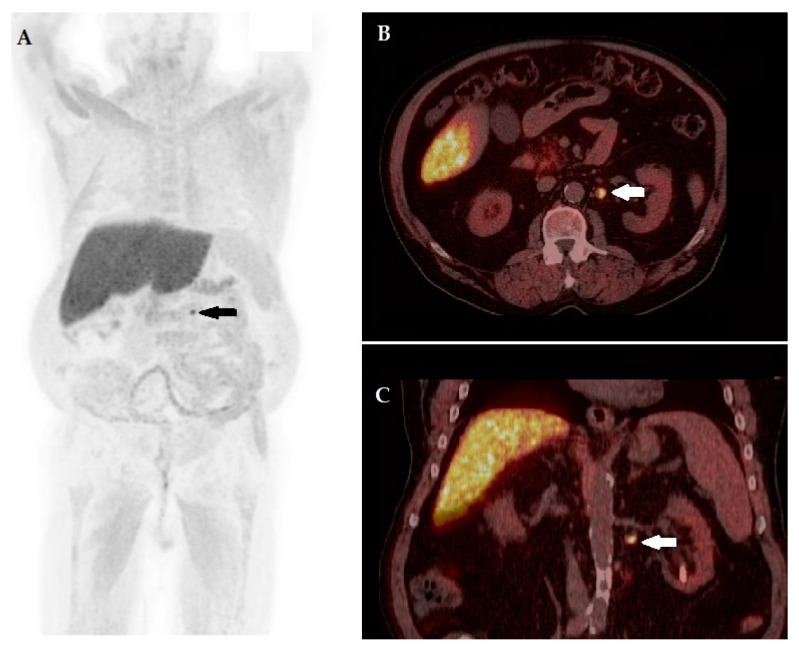
A 74-year-old patient 5 years post radiotherapy for cT2b ISUP Grade Group 4 prostate adenocarcinoma, presenting biochemical recurrence with PSA level prior to PET/CT of 1.25 ng/mL. (**A**) MIP image showing an area of increased tracer incorporation in the left umbilical region (arrow). Fused corresponding PET/CT axial (**B**, arrow) and coronal (**C**, arrow) slices depicting a round-shaped para-aortic lymph node, located below the left kidney artery, characterized by ^18^F-FACBC pathological uptake, with a maximum diameter of 14 mm and a maximum standardized uptake value (SUV_max_) of 10.1, superior to bone marrow SUV_mean_ (3.3). Final diagnosis was T0N0M1a according to PROMISE. Intended therapy prior to PET/CT was androgen deprivation therapy, after the collegial discussion of ^18^F-FACBC PET/CT’s results, the patient was submitted to stereotactic radiotherapy on the abdominal node with PSA response.

Figure 6 and Figure 7 show emblematic cases of patients with PC BCR, in which ^18^F-FACBC PET/CT determined significant changes in management.

According to the defined criteria, PET/CT’s impact on clinical management was considered adequate in 29 out of 33 cases (87.8%).

## 4. Discussion

Our retrospective analysis indicates meaningful DR (i.e., 76.9%) and a positive predictive value (i.e., 96.7%) for ^18^F-FACBC PET/CT in patients with BCR from PC. These data are substantially in line with those reported in a previously published meta-analysis encompassing nine clinical studies, indicating at per-patient analysis a sensitivity and specificity of 86.3% and 75.9%, respectively, for PC recurrence detection [18]. Of note, our results show that ^18^F-FACBC PET/CT may also represent a valuable tool in patients with low (0.58–0.9 ng/mL) or very low (0.2–0.57 ng/mL) PSA levels, with DR of 71.4% and 66.7%, respectively.

Although ^18^F-FACBC PET/CT has been implemented in clinical practice in many centers, its DR in subjects with low PSA has not been fully investigated yet. The results of the FALCON clinical trial, which collected patients from six different UK centers and aimed to assess ^18^F-FACBC PET/CT’s impact in patients with BCR from PC after radical treatment, found positive lesions in 58 out of 104 subjects (DR = 56%), mainly located in the prostate bed and pelvic lymph nodes. Of note, DR strongly correlated with PSA levels, since lymph node and skeletal positivity ranged from 8.9% and 3.6%, respectively, at PSA ≤ 1 ng/mL, to 50% and 13%, respectively, at >10 ng/mL [19]. Furthermore, the authors registered a meaningful impact of PET/CT on patients’ clinical management, with the major changes being the switch from an intended systemic/salvage therapy to watchful waiting (24%) and from salvage therapy to systemic therapy (24%).

Similar results have been obtained in the clinical trial LOCATE, including a larger number of patients (n = 213) collected from 15 US centers: the authors report for ^18^F-FACBC a DR of 57% in the whole cohort, with an increasing sensitivity for higher PSA values. It is worth mentioning that DR resulted in 31% and 50% among patients with PSA values ranging 0–0.5 ng/mL and 0.5–1 ng/mL, respectively [20]. It has to be underlined that PET/CT led to a major change in patients’ management in 126 subjects (59%) with a major impact in 98 cases (78%).

In a retrospective multicenter study, including a large cohort (n = 596), ^18^F-FACBC PET/CT’s DR was 67.7%, with prostate bed and pelvic lymph nodes being the most frequent sites of disease localization. Of note, DR was meaningfully lower (41.4%) in subjects in the lowest quartile of PSA levels (i.e., <0.79 ng/mL). If has to be underlined that, among the enrolled patients, 143 (23.9%) PET/CT scans were correlated with histology as a standard of truth [21].

These preliminary data [19,20,21] suggest a low DR of ^18^F-FACBC for the detection of BCR at low PSA levels and are partially in disagreement with other recently published papers. Armstrong et al. retrospectively analyzed 115 patients with BCR submitted to ^18^F-FACBC and stratified results by different intervals of PSA: at PSA levels of less than 0.5, 0.5 to 2.0, and greater than 2, lesions were detected in 55.5%, 70.6% and 91.5% of patients, respectively [22]. More recently, a further retrospective study from Marcus and colleagues assessed ^18^F-FACBC PET/CT’s DR in a group of patients presenting very low PSA levels (i.e., ≤0.3 ng/mL), reporting positivity rates of 43.8%, 60.0% and 65.2% for PSA < 0.1 ng/mL, 0.1–<0.2 ng/mL and 0.2–≤0.3 ng/mL, respectively [23].

Our data are substantially in line with those published by Marcus’s and Armstrong’s group. Although ^18^F-FACBC PET/CT showed an increased DR with increasing PSA values and, in fact, PSA values were significantly different among patients with positive and negative scans, it was demonstrated to also be effective in detecting PC recurrence in half of our patients (51.8%) presenting PSA values < 1 ng/mL.

The discrepancies of results among different research groups might be explained by several factors. First of all, patient selection might play a relevant role. It is still debated, in fact, whether or not ongoing ADT might influence PET/CT’s sensitivity [24]. In our cohort, we decided to exclude patients ongoing ADT, according to the previously performed clinical trial FALCON [19], in order to avoid a potential inference of anti-androgen treatment on ^18^F-FACBC PET/CT’s DR. Second, histology was only available in a small number of papers as a standard of truth. However, it has to be underlined that biopsy for recurrence verification or histology after the “so-called” salvage-surgery is not common in clinical practice and is not exempt from controversy [25]; therefore, in real-life practice the reference is often represented by clinical evolution or confirmatory imaging.

Another relevant issue to be considered is related to differences in available technologies for PET/CT acquisition. Preliminary data have indicated that, regarding PET/CT with ^68^Ga-PSMA-11, DR is strongly influenced by the technology used [26,27]. In our study, two highly performing PET/CT devices (i.e., one equipped with last-generation photomultiplier tubes/PMT and the other with silicon photomultipliers/SIMPs) were applied, and in both facilities, the algorithm for image reconstruction applied Time of Flight (TOF) information to improve localization of the annihilation events thus increasing the signal-to-noise ratio. Further studies are needed to better define the degree to which technological improvements may positively influence ^18^F-FACBC PET/CT’s diagnostic performance.

In our cohort of patients, we classified PET/CT images through the TNM-based PROMISE reading system, which was developed by Eiber et al. [17] for PSMA-PET [28], but can be easily adapted to ^18^F-FACBC PET/CT, as previously demonstrated by Salavati et al. [29]. We found PROMISE feasible and easy to apply for the classification of PET/CT images, and among our patients, the majority (i.e., 59.3%) were, at final diagnosis, TrN1. These results are consistent with tracer biodistribution and its minimal or absent accumulation in bladder, allowing the detection of pathological lesions in pelvis. From this perspective, ^18^F-FACBC PET/CT might offer advantages with respect to some PSMA-ligands, such as ^68^Ga-PSMA-11, which presents meaningful bladder accumulation potentially harming the detection of pathological focuses in pelvis, therefore requiring specific protocols, such as forced diuresis and late imaging [30]. In this regard, it is worth mentioning a recently published meta-analysis comparing the 3 available radiopharmaceuticals (i.e., ^18^F-choline, ^18^F-FACBC and ^68^Ga-PSMA-11) for the detection of PC BCR [31]: the authors collected and evaluated an overall number of 46 studies and registered a DR of 66%, 74% and 83% for ^18^F-choline, ^18^F-FACBC and ^68^Ga-PSMA-11, respectively. Of note, ^68^Ga-PSMA-11 substantially outperformed the other two tracers in patients presenting PSA level < 1 ng/mL. Nevertheless, it has to be underlined that in the aforementioned study [31], the authors did not take into account the more recently published studies highlighting the meaningful ^18^F-FACBC PET/CT’s DR for low and very low PSA values [13,22,29].

A further consideration has to be made regarding ^18^F-FACBC PET/CT’s impact on patients’ clinical management. Our results are substantially in line with those previously reported [19,20], indicating a significant contribution of PET/CT in patients’ therapeutic work-flow, particularly with respect to the use of PET-directed radiotherapeutic treatments that can be successfully applied, on the one hand, in order to prolong survival and, on the other hand, to positively influence subjects’ quality of life, delaying, in some cases, the use of ADT with the consequent effects on erectile function [14]. In our study, we enrolled patients with BCR as defined according to the Phoenix criteria [15], which are commonly applied in clinical practice. In this regard, the recently published retrospective analysis performed by Salavati and coworkers [29] must be cited, which assessed the potential of ^18^F-FACBC PET/CT in patients with BCR and PSA levels less than those fixed by the Phoenix criteria. The authors reported a DR of 79% in the whole cohort and 62% in subjects with PSA values below the aforementioned criteria. Of note, in line with our analysis, neither pre-scan PSA nor PSA DT were found to present a significant predictive value on a positive ^18^F-FACBC PET/CT scan.

Our study presents several limitations: first of all, as previously stated, the lack of histological confirmation. Furthermore, the retrospective nature of the study might have introduced bias in patients’ selection and data interpretation. Finally, another limitation is represented by the relatively small number of subjects in our analysis, although not meaningfully different from that included in other published papers. However, also taking into account the aforementioned limitations, given the relatively few studies focusing on ^18^F-FACBC PET/CT’s sensitivity in patients with BCR at different PSA levels, we think that our data might be worthy of consideration.

## 5. Conclusions

Our results indicate that ^18^F-FACBC PET/CT may also represent a useful diagnostic tool in patients with BCR at low PSA levels, with meaningful impact on patients’ clinical management. Further studies with large cohorts, collected through multicenter cooperation, are needed to better define how much patients’ selection and technological facility might impact ^18^F-FACBC PET/CT’s diagnostic performance.

## Figures and Tables

**Table 1:** Summary of patients’ clinical-demographic features.

**Age (years)**		**-**
median	74	
mean ± SD	73.2 ± 6.9	
**Initial Therapy**	**n**	**%**
Surgery only	45	55.5
Radiation therapy only	20	24.7
Surgery + radiation therapy	14	17.3
Brachytherapy	2	2.4
**ISUP**	**n**	**%**
ISUP 1	8	9.9
ISUP 2	8	9.9
ISUP 3	27	33.3
ISUP 4	33	40.7
ISUP 5	5	6.2
**Time from Diagnosis to Relapse (y)**		
median	7	
mean ± SD	7 ± 4.3	
**PSA prior to PET/CT**		
median	0.99	
mean ± SD	1.27 ± 1.1	
**PSA doubling time**	n	%
<6 months	23	28.4
>6 months	58	71.6

Abbreviations: y—years; ISUP—International Society of Urological Pathology (ISUP) Grade Group, PSA—prostate specific antigen.

**Table 2:** PET/CT results classified according to PROMISE applied to ^18^F-FACBC.

Final Classification	TNM
Local relapse (Tr/N1) n = 48 (59.3%)	TrN0M0, n = 25 (30.8%)
	T0N1M0, n = 16 (19.8%)
	TrN1M0, n = 7 (8.7%)
Distant Lymph nodes (M1a) n = 8 (9.9%)	T0N0M1a, n = 3 (3.8%)
	TrN0M1a, n = 1 (1.2%)
	T0N1M1a, n = 3 (3.8%)
	TrN1M1a, n = 1 (1.2%)
Bone/visceral metastases (M1b/C) n = 6 (7.4%)	TrN0M1b, n = 4 (4.9%)
	T0N0M1b, n = 1 (1.2%)
	M1c, n= 1 (1.2%)
Negative n = 19 (23.4%)	T0N0M0

## Data Availability

Dataset used or analyzed during the current study are available from the corresponding author on reasonable request.

## Supplementary Materials

The following supporting information can be downloaded at: https://www.mdpi.com/article/10.3390/biomedicines10010177/s1. Supplemental data 1.
