# Supplementary material for: Detection Rate and Clinical Impact of PET/CT with 18F-FACBC in Patients with Biochemical Recurrence of Prostate Cancer: A Retrospective Bicentric Study
Source: Biomedicines. 2022 Jan 15;10(1):177. doi: 10.3390/biomedicines10010177 (PMC8773479; doi:10.3390/biomedicines10010177)
Supplement: Supplementary file 1 [file biomedicines-10-00177-s001.zip › biomedicines-1542963-supplementary.pdf]

biomedicines

MDPI

Supplemental data 1

PET/CT scans in Latina (S.M. Goretti Hospital) were performed with a digital Biograph Vision PET/CT system (Siemens Healthcare; Erlangen, Germany). A CT scan from proximal thigh to skull base was performed with slice thickness of 1.0 mm, pitch factor 1, bone and soft tissue reconstruction kernels and maximum of 120 keV and 90 mAs by applying CARE kV and CARE Dose. After CT scanning, a whole body PET (proximal thigh to skull base) was acquired at 3-5 min post tracer administration in 3D (matrix: 440 × 440) with a zoom factor of 1.0. Digital PET acquisitions were acquired on a Siemens Biograph Vision 450 with an axial FOV of 197 mm using continuous-bed motion (FlowMotion®) with a bed speed of 1.5 mm/s (equivalent to approximately 90s/bed position). Reconstruction was conducted with a TrueX + TOF algorithm and Gauss-filtered to a transaxial resolution of 2 mm at FWHM (full width at half maximum). Attenuation correction was performed using the low dose non-enhanced computed tomography data.

PET/CT scans in Trieste (Cattinara University Hospital) were performed with the GE DMI-DR PET/CT system (General Electric Healthcare System, Milwaukee, WI, USA), a PMT-based device equipped with the LightBurst PET detectors and a 128-slice CT system. As previously described, CT was performed from proximal thigh to skull base, followed by whole body (proximal thigh to skull base) PET scan at 3-5 min post ^{18}F-FACBC intravenous administration. The DMI-DR AFOV and TFOV were equal to 15.6 cm and 70 cm, each ring consisting of 24 detectors. Reconstructions were performed using the VPHD (3D OSEM) and VPHD-S (3D OSEM + PSF) algorithms in a 384 × 384 × 71 matrix with 4 iterations, 24 subsets, and a 2.0-mm Gaussian filter. Attenuation correction was performed using the low dose non-enhanced computed tomography data.
